# Postoperative drainage after pancreatoduodenectomy: a randomized controlled trial among patients with intermediate and low risks for pancreatic fistula—DRAIN1

**DOI:** 10.1186/s13063-026-09762-9

**Published:** 2026-05-05

**Authors:** Sebastian Wallon, Caroline Williamsson, Victor Karlsson, Johanna Wennerblom, Svein-Olav Bratlie, Per Sandström, Bobby Tingstedt, Bergthor Björnsson

**Affiliations:** 1 Department of Biomedical and Clinical Sciences, Linköping University, Linköping, Sweden; 2 Department of Surgery, Region Östergötland, Linköping, Sweden; 3 Department of Clinical Sciences Lund, Surgery, Lund University and Skåne University Hospital, Getingevägen 4, Lund, 221 85 Sweden; 4 Department of Surgery, Sahlgrenska University Hospital, Gothenburg, Sweden

**Keywords:** Pancreatoduodenectomy, Intra-abdominal drain, Whipple procedure, Postoperative pancreatic fistulae

## Abstract

**Background:**

Routine use of surgical drains after abdominal operations has largely been abandoned over the past decades. Studies have failed to demonstrate benefits of routine drainage following liver, gallbladder, gastric, and colorectal surgeries.

Until recently, intraoperative placement of abdominal drains was the gold standard in pancreatoduodenectomies (PDs) due to concerns about uncontrolled postoperative pancreatic fistula (POPF). A large randomized trial in 2014 reported increased mortality in patients without postoperative drain placement. However, as the study did not stratify participants based on their preoperative risk of developing a POPF, further research is needed.

Limited evidence from a non-randomized cohort suggests that omitting drains may be safe in very low-risk settings. However, a larger comparative study, including a broader range of PD cases, is necessary to confirm these findings.

**Methods:**

This is a two-arm, randomized, controlled, non-blinded, multicenter trial comparing intra-abdominal drain placement with no drain placement during planned pancreatoduodenectomies (PDs).

Eligible patients who meet the inclusion criteria will be assessed for their individual risk of postoperative pancreatic fistula (POPF) using a risk scoring system. They will then be randomized into either the drain placement or no drain placement group.

The groups will be compared using the chi-square test for categorical variables and Fisher’s exact test. Logistic regression models will be used to calculate odds ratios for morbidity. Univariable and multivariable models will assess the impact of drain placement on clinical outcomes.

**Discussion:**

This trial aims to determine whether omitting routine intraoperative drain placement reduces the risk of complications in patients undergoing pancreatoduodenectomy (PD). It will provide level 1 evidence on the association between routine intra-abdominal drainage and postoperative complications in patients with a low to intermediate risk of developing a postoperative pancreatic fistula (POPF). The findings will contribute to future treatment guidelines by expanding the available knowledge on optimal drainage strategies.

**Trial registration:**

ClinicalTrials.gov Identifier: NCT05270564. Registered on February 16 2022.

**Supplementary Information:**

The online version contains supplementary material available at 10.1186/s13063-026-09762-9.

## Administrative information

Note: the numbers in curly brackets in this protocol refer to SPIRIT checklist item numbers. The order of the items has been modified to group similar items (see http://www.equator-network.org/reporting-guidelines/spirit-2013-statement-defining-standard-protocol-items-for-clinical-trials/).

**Table:** 

Title {1}	Postoperative drainage after pancreatoduodenectomy: a randomized controlled trial among patients with intermediate and low risks for pancreatic fistula
Trial registration {2a and 2b}	ClinicalTrials.gov Identifier: NCT05270564
Protocol version {3}	Version 1.1, published in primary registry on March 8th 2022
Funding {4}	The study was initiated by the researchers and no specific funding has been obtained.
Author details {5a}	Sebastian Wallon,1,2*, Caroline Williamsson3*, Victor Karlsson1,2, Johanna Wennerblom4, Svein-Olav Bratlie4, Per Sandström1,2, Bobby Tingstedt3#, Bergthor Björnsson1,2# 1 Department of Biomedical and Clinical Sciences, Linköping University, Linköping, Sweden. 2 Department of Surgery, Region Östergötland, Linköping Sweden. 3 Department of Clinical Sciences Lund, Surgery, Lund University and Skåne University Hospital, Getingevägen 4, 221 85, Lund, Sweden. 4 Department of Surgery, Sahlgrenska University Hospital, Gothenburg, Sweden. *These authors share first authorship # These authors share senior authorship
Name and contact information for the trial sponsor {5b}	Skane University Hospital, Getingevägen 4, 221 85, Lund, Sweden. stefan.santen@skane.se
Role of sponsor {5c}	The sponsor played no part in study design; collection, management, analysis, and interpretation of data; writing of the report; and the decision to submit the report for publication

## Introduction

### Background and rationale {6a}

The routine placement of surgical drains following abdominal operations has largely been abandoned over the past decades. Previous studies have failed to demonstrate benefits of routine drainage following liver [1], gallbladder [2], gastric [3], and colorectal surgery [4, 5]. However, intraoperative placement of an abdominal drain remains the gold standard in pancreatoduodenectomies (PDs) due to concerns about uncontrolled postoperative pancreatic fistula (POPF). Uncontrolled POPF can have severe consequences, including intra-abdominal abscesses and post-pancreatectomy hemorrhage (PPH), which may lead to mortality. The rationale for drain placement is to control and remove collected fluid, potentially mitigating the impact of a fistula if one develops. However, the routine use of drains has been increasingly questioned.

Large retrospective cohort studies [6–10], a single-institution randomized controlled trial (RCT) [11], and multiple meta-analyses [12–15] have failed to show a clear benefit of routine drainage. Some reports suggest that patients without routine drainage experience lower rates of wound infection and delayed gastric emptying [8], as well as reduced hospital readmission rates [9]. Additionally, routine drainage has been associated with prolonged hospital stays [6, 9].

More recently, a non-randomized sub-cohort of PD patients with a very low risk of POPF, as determined by the fistula risk score [16], has been managed without drains or with early drain removal, without subsequent fistula development [17]. Furthermore, multiple studies have shown significantly lower rates of POPF and shorter median hospital stays in patients randomized to drain removal on postoperative day (POD) 3 compared to POD 5 [18, 19]. Additionally, delayed drain removal appears to increase postoperative morbidity, as higher rates of intra-abdominal infections have been observed in patients whose drains were removed on POD 8 compared to POD 4 [20].

To date, available evidence has primarily focused on the safety of a no-drain policy or early drain removal. In line with recent data demonstrating the superiority of a no-drain approach in left-sided pancreatic resections, this question needs further exploration in the context of PD [21]. The risk of POPF after PD is well known to correlate with pancreatic remnant characteristics, with soft, fatty glands and small duct diameters carrying the highest risk [22, 23]. Additionally, patient factors such as body mass index (BMI) and comorbidities have been linked to a higher risk of fistula formation [24, 25], as reflected in various POPF predictive scores [26, 27]. A preoperative risk score, incorporating pancreatic duct diameter and BMI, was developed by Roberts et al. [25] to aid in patient selection.

Calls for a large, randomized trial have been made, and in 2014, a multicenter study reported higher mortality among patients who did not receive drains [28]. However, this study did not account for variations in POPF risk based on preoperative or intraoperative predictors. Patients were included regardless of whether they had a high, intermediate, or low risk of developing POPF. Notably, among the 10 fatalities in the study, 8 had a soft pancreas, and 6 had a pancreatic duct diameter smaller than 3 mm. In their conclusion, the authors emphasized the need for studies incorporating predictive factors to identify patients who may safely undergo PD without routine drainage.

For this reason, the current RCT aims to evaluate the role of surgical drains in patients with a low or intermediate risk of developing pancreatic fistulae.

### Objectives {7}

The study hypothesis is that omitting intraoperatively placed drains decreases the morbidity rate after PD in patients with low to intermediate risk of POPF.

### Trial design {8}

DRAIN1 is a two-armed, randomized, controlled, non-blinded, multicenter trial.

## Methods: participants, interventions, and outcomes

### Study setting {9}

The DRAIN1 trial is conducted in (academic) university hospitals in Sweden. A list of study sites can be obtained from ClinicalTrials.gov (NCT05270564).

### Eligibility criteria {10}

#### Inclusion criteria

Patients must meet the following criteria to be included in the study:

1. Undergoing pancreatoduodenectomy (PD), regardless of sex.
2. Predicted pancreatic fistula risk score of ≤ 10, based on the predictive score of Roberts et al. that considers only body mass index and pancreatic duct width [25].
3. Age ≥ 18 years.
4. Provision of written informed consent.
5. Expected survival time of more than 6 months.

#### Exclusion criteria

Patients meeting any of the following criteria will be excluded:

1. Predicted high risk of postoperative pancreatic fistula (i.e., risk score > 10, according to Roberts et al. [25]).
2. Presence of intra-abdominal abscess or infection.
3. American Society of Anesthesiologists (ASA) score > 3.
4. Pregnancy.
5. Expected lack of compliance.

#### Eligibility criteria for participating centers

Centers must have a dedicated pancreatic surgery unit performing at least 40 PDs per year to be eligible for participation.

### Who will take informed consent? {26a}

Written informed consent is obtained by surgeons participating in pancreatic surgery and perioperative treatment of the actual patients.

### Additional consent provisions for collection and use of participant data and biological specimens {26b}

The consent form does not ask participants whether they agree to the use of their data if they choose to withdraw from the trial. Consequently, data from patients who withdraw will not be included in the analysis. Additionally, participants are not asked for permission to allow the research team to share relevant data with other researchers or regulatory authorities. This trial does not involve the collection or storage of biological specimens.

## Interventions

### Explanation for the choice of comparators {6b}

The primary endpoint, surgery-related morbidity, was chosen as the main comparator because morbidity in pancreatic surgery presents a significant burden for patients and may potentially hinder additional systemic treatment when indicated.

### Intervention description {11a}

All patients will undergo pancreatoduodenectomy (PD) according to the local standards of each participating site. In the treatment group (no drain), patients will not receive a drain intraoperatively, while the control group (traditional treatment) will receive a drain at the end of the surgical procedure. The type of drain and its postoperative management will be determined by each center’s standard practices. Adherence to the study protocol will be ensured by treating all included patients as intended in both the treatment and control groups, and by providing appropriate follow-up as outlined in the study protocol.

### Criteria for discontinuing or modifying allocated interventions {11b}

If the operating surgeon deems a drain necessary during the procedure, the patient will be removed from the allocated group and analyzed separately, in addition to intention-to-treat and per-protocol analyses. The decision to place a drain despite the patient meeting preoperative inclusion criteria and being randomized to the no-drain group is based solely on the surgeon’s clinical judgment rather than predefined criteria.

### Strategies to improve adherence to interventions {11c}

As the intervention is an intraoperative drain placement or not, no continuous measures are available or needed in order to adhere to interventions for individual patients.

### Relevant concomitant care permitted or prohibited during the trial {11d}

Participation in DRAIN1 does not influence other parts of the medical and surgical care of the participants.

### Provisions for post-trial care {30}

No provision is provided for trial participation; the participants are incurred by Swedish health care insurance.

### Outcomes {12}

#### Primary endpoint

The primary endpoint of the study is the overall morbidity rate, classified according to the Clavien–Dindo classification. The study hypothesis is that omitting intraoperatively placed intra-abdominal drains reduces the morbidity rate. Given the significant morbidity associated with pancreatic surgery, which may hinder the administration of adjuvant chemotherapy when indicated, this endpoint is clinically well-justified.

#### Secondary endpoints

1. Mortality rate: defined as in-hospital mortality, 30-day mortality, and 90-day mortality.
2. Severe morbidity rate: classified as Clavien–Dindo grade > 3a.
3. POPF rate: defined according to the ISGPS criteria [29].
4. Intra-abdominal abscess rate: defined as a fluid collection with infectious signs on radiological imaging.
5. Wound infection rate: defined by clinical signs such as redness or purulent discharge.
6. Delayed gastric emptying rate: classified according to the ISGPF definition [30].
7. Post-pancreatectomy bleeding rate: defined according to the ISGPF criteria [31].
8. Rate of interventional radiology procedures: defined as interventions requiring radiologic guidance, such as ultrasound-guided drain placement or angiographic procedures.
9. Need for reoperation.
10. Length of hospital stay (in days).
11. Readmission rate within 90 days.

The study results will be submitted for publication upon completion, regardless of the magnitude of the findings.

### Participant timeline {13}

**Fig. 1:**
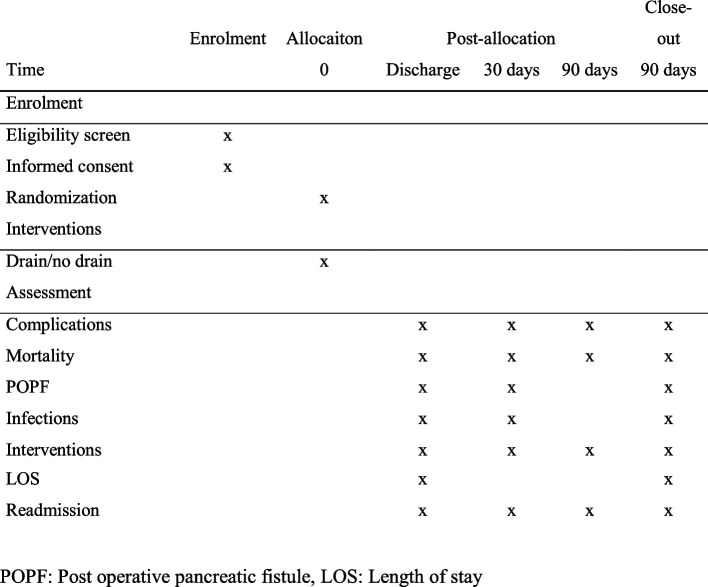
Overview of study procedures. POPF, postoperative pancreatic fistula, LOS, length of stay

Figure 1 shows the intended timeline of the participating patients, from enrollment to close-out.

### Sample size {14}

The sample size calculation is based on the primary endpoint: the complication rate classified according to the Clavien–Dindo classification. The study is powered to detect a reduction in overall morbidity from 50 to 35%, with 80% power and a 5% two-tailed significance level [32]. The estimate is based on previous publications [6, 8]. To achieve this, a total of 133 patients per group is required. To account for an anticipated 10% dropout and loss to follow-up rate, a total of 300 patients will be included in the study.

### Recruitment {15}

To enhance the screening process for study enrollment, the pancreatic duct diameter is included as a standard measurement in tumor board discussions at all participating sites. Additionally, the involvement of research nurses in tumor boards will help ensure the identification of eligible participants.

## Assignment of interventions: allocation

### Sequence generation {16a}

Randomization will be conducted at each participating center by a research nurse using a computer-generated sequence with blocks of eight. Block randomization ensures balance between treatment groups and will be implemented at each site, with blocks of eight distributed accordingly. The research nurse responsible for randomization will not be involved in patient care to maintain allocation integrity. Since participating centers routinely perform either pancreatojejunostomy or pancreatogastrostomy as their standard reconstruction method, stratification for this factor is not required.

### Concealment mechanism {16b}

Allocation concealment will be ensured using opaque, identical envelopes for all groups. Patients preoperatively enrolled in the study will be randomized prior to surgery but allocation is done in the operating theater at the time the decision to proceed with resection is made. This is done to reduce dropouts due to non-resections and to allow for an “opt out” in case of unexpected intraoperative findings.

### Implementation {16c}

The randomization will be performed by a research nurse who will not participate in patient care, patient allocation is done in the operating room by the leading surgeon.

## Assignment of interventions: blinding

### Who will be blinded {17a}

The DRAIN1 trial is an open-label trial.

### Procedure for unblinding if needed {17b}

As blinding is not applied, no unblinding procedures are included.

## Data collection and management

### Plans for assessment and collection of outcomes {18a}

Data is collected locally for the baseline variables as well as the outcomes as stated above. The data collection is done by surgeons and research nurses. The outcomes will be assessed as described below.

### Plans to promote participant retention and complete follow-up {18b}

Due to the short follow-up period (90 days), complete follow-up is anticipated without the need for specific retention measures. Since the intervention occurs as a single event during surgery, the dropout rate is expected to be low. Additionally, as the informed consent does not request permission to use previously collected data or gather additional data upon withdrawal, no specific plan exists for handling such data.

### Data management {19}

The data collected is entered in an electronic database that includes range checks as indicated; double entry will not be used.

### Confidentiality {27}

Every patient enrolled in the study is given a pseudonym and the code list for them is kept locked and only accessible to the involved researchers. The presentation of the results will be done for groups; thus, individual identities will not be revealed.

### Plans for collection, laboratory evaluation and storage of biological specimens for genetic or molecular analysis in this trial/future use {33}

No biological specimens or material is collected in the DRAIN1 trial.

## Statistical methods

### Statistical methods for primary and secondary outcomes {20a}

The statistical analysis will follow the intention-to-treat principle. Baseline characteristics will be presented as means with standard deviations or medians with interquartile ranges, depending on data distribution. The chi-square test will be used for categorical variables when the expected count is ≥ 5; otherwise, Fisher’s exact test will be applied. Logistic regression models will be used to estimate odds ratios for morbidity risk and drain use. Univariable and multivariable models will be employed to assess the impact of drain placement on outcomes.

### Interim analyses {21b}

A safety data monitoring board will oversee patient outcomes and conduct an interim analysis. The study will be halted if severe morbidity (Clavien–Dindo > 3a) or mortality exceeds 50% of expected rates. The board will consist of two independent experts from different centers who are not involved in the study. They will communicate directly with the primary investigator about the timing and conduction of the interim analysis.

### Methods for additional analyses (e.g., subgroup analyses) {20b}

The planned subgroup analysis to be performed is a separate analysis of the primary end-point in patients having pancreatojejunostomy and pancreatogastrostomy, respectively. 

### Methods in analysis to handle protocol non-adherence and any statistical methods to handle missing data {20c}

Patients allocated to no-drain treatment but receiving a drain will be separately analyzed in addition to those being included in the intention-to-treat analysis of that group and the group also being analyzed in a per-protocol analysis. Missing data will not be imputed.

### Plans to give access to the full protocol, participant-level data, and statistical code {31c}

The full dataset will not be made public but may be available upon contact with the principal investigator.

## Oversight and monitoring

### Composition of the coordinating center and trial steering committee {5d}

The coordinating center is responsible for both local data collection and compiling data from all participating centers. The trial steering committee is also the project management group and includes a local principal investigator (PI) from each participating center to ensure continuous support and facilitate information exchange between sites. The committee meets twice per year, with additional meetings scheduled as needed. If the safety data monitoring board raises concerns, these will be addressed in an additional meeting of the trial steering committee.

### Composition of the data monitoring committee, its role, and reporting structure {21a}

The data monitoring committee consists of two experienced pancreatic surgeons from independent, non-participating centers. Severe complications are reported to the board within 72 h of identification, along with the updated number of enrolled patients. The board evaluates these cases as outlined in the “Interim Analyses” section and provides a recommendation to the principal investigator (PI) regarding whether the study should be continued or halted if necessary.

### Adverse event reporting and harms {22}

Adverse events related to study participation will be reported upon publication of the results, as they constitute both the primary endpoint and a significant portion of the secondary endpoints. Morbidity is also closely monitored by the data monitoring board to ensure the trial is conducted safely. To facilitate this, complications classified as Clavien–Dindo grade > 3a are reported to the board within 72 h.

### Frequency and plans for auditing trial conduct {23}

No audit, other than the continuous reporting of severe complications as described above, is planned in the DRAIN1 trial.

### Plans for communicating important protocol amendments to relevant parties (e.g., trial participants, ethical committees) {25}

All protocol amendments will be discussed in the trial steering committee, and upon amendment of the ethical approval (if necessary), the new protocol will be distributed to each trial site after informing the sponsor. If a minor amendment that does not require amendment of the ethical approval is made, the distribution follows the same route. Major revisions of the protocol will be updated in the clinical trial registry.

### Dissemination plans {31a}

Regardless of outcome, the results will be submitted for publication in a peer-reviewed medical journal.

## Discussion

The current trial will be the first randomized controlled trial conducted to determine whether omitting routine intraoperative drain placement in low- and intermediate-risk patients undergoing pancreatoduodenectomy reduces complication rates. A previous multicenter RCT failed to address this specific question due to the inclusion of high-risk patients for postoperative pancreatic fistula development. Due to the discouraging results of that study, routine drain placement remains the standard practice in PD.

The primary concern justifying routine drain placement has been the risk of developing an uncontrolled POPF. However, with improved risk stratification, it has become evident that patients have varying risks of developing POPF. Some centers have already adopted a no-drain policy in cases with a very low risk of POPF, despite the lack of high-level evidence. Since drain placement in PD may be associated with increased rates of certain postoperative complications, further investigation is necessary to determine whether omitting routine drains is beneficial for specific patient subgroups.

Given the previously reported higher mortality in patients who did not receive drains after PD [28], this trial will utilize preoperative risk assessment to exclude patients at high risk for POPF.

A major strength of the current study is its multicenter design, which will enhance the generalizability of findings and improve the likelihood of reproducibility in different settings. Additionally, the sample size calculation is based on robust nationwide registry data with high coverage, reflecting real-world experience in pancreatic surgery.

However, certain limitations must be considered. First, the lack of standardization in surgical indications, procedures, and postoperative treatments introduces heterogeneity. While this may improve external validity, it also increases susceptibility to bias. Second, the COVID-19 pandemic reduced the enrollment rate, which may affect results due to evolving treatment approaches for pancreatic head tumors. The superiority design of the trial could be questioned given the perceived benefits with no drain for a patient. However, when this trial was designed there was a strong drain policy in most pancreatic centers and a non-inferiority design was deemed too weak for clinically meaningful results. The final allocation of study participants in the operating room may induce a selection bias as events during surgery might prevent surgeons from enrolling certain patients. This was included in the study plan as a safety measure and reasons for not allocating patients as well as their outcomes were documented.

Another limitation is the absence of standardized early drain removal in the control group. However, this is unlikely to impact the study results significantly, as participating centers have adhered to early drain removal protocols for over a decade when no POPF signs are present. Additionally, since the trial was designed nearly a decade ago, newer predictive models for POPF have emerged. The selected model may be subject to criticism, as some may argue that other models are more relevant.

Lastly, at the time of trial initiation, minimally invasive PD was not performed in Sweden and was therefore not included in the protocol.

Despite these limitations, the study is expected to provide valuable insights into the necessity of routine drain placement in patients with low to intermediate risk for POPF. The findings may contribute to a shift in clinical practice, leading to more patients being treated without routine intraoperative drain placement.

## Trial status

The trial is currently still recruiting patients as of March 2025. The current protocol version is 1.0 and no changes are planned. Randomization and recruitment began in May 2016, and to date, 220 (73%) patients have been included. The study is behind schedule due to the COVID-19 pandemic and due to problems recruiting additional centers, but the inclusion rate has increased again and is expected to be completed by the end of 2026.

## Supplementary Information

Supplementary Material 1. SPIRIT checklist.

## Data Availability

Datasets are not available due to ongoing recruitment. BB and BT will have access to the full dataset that may be available upon request.

## Abbreviations

PD: Pancreatoduodenectomy
POPF: Postoperative pancreatic fistula
SPIRIT: Standard Protocol Items: Recommendations for Interventional Trials
RCT: Randomized controlled trial
ISGPS: International study group for pancreatic surgery
